# Maternal intake of sugar during pregnancy and childhood respiratory and atopic outcomes

**DOI:** 10.1183/13993003.00073-2017

**Published:** 2017-07-06

**Authors:** Annabelle Bédard, Kate Northstone, A. John Henderson, Seif O. Shaheen

**Affiliations:** 1 Centre for Primary Care and Public Health, Barts and The London School of Medicine and Dentistry, Queen Mary University of London, London, UK; 2 National Institute for Health Research Collaboration for Leadership in Applied Health Research and Care West, Bristol, UK; 3 School of Social and Community Medicine, University of Bristol, Bristol, UK; 4 Joint senior authors

## Abstract

The possible role of maternal consumption of free sugar during pregnancy in the inception of respiratory and atopic diseases has not been studied. We aimed to study the relationship between maternal intake of free sugar during pregnancy and respiratory and atopic outcomes in the offspring in a population-based birth cohort, the Avon Longitudinal Study of Parents and Children.

We analysed associations between maternal intake of free sugar in pregnancy (estimated by a food frequency questionnaire), and current doctor-diagnosed asthma, wheezing, hay fever, eczema, atopy, serum total IgE and lung function in children aged 7–9 years (n=8956 with information on maternal diet in pregnancy and at least one outcome of interest).

After controlling for potential confounders, maternal intake of free sugar was positively associated with atopy (OR for highest *versus* lowest quintile of sugar intake 1.38, 95% CI 1.06–1.78; per quintile p-trend=0.006) and atopic asthma (OR 2.01, 95% CI 1.23–3.29; per quintile p-trend=0.004). These associations were not confounded by intake of sugar in early childhood, which was unrelated to these outcomes.

Our results suggest that a higher maternal intake of free sugar during pregnancy is associated with an increased risk of atopy and atopic asthma in the offspring, independently of sugar intake in early childhood.

## Abstract 2

**Higher maternal intake of sugar in pregnancy may increase the risk of allergy and allergic asthma in the offspring** http://ow.ly/zehc30bFswP

## Introduction

There has been considerable interest in the role of maternal diet in pregnancy in the aetiology of childhood asthma and atopy [1]. Studies have focused particularly on the potentially beneficial effects of antioxidants, following the hypothesis that a declining intake of antioxidants in Westernised countries has led to a reduction in pulmonary antioxidant defences, and hence to an increase in prevalence of asthma and atopy in recent decades [2]. An alternative hypothesis, which has received less attention, is that the epidemic of asthma and atopy in the West could partly be explained by an increasing dietary intake of foods and constituents which may be harmful. Between 1970 and 2000, there was a 25% increase in the per capita consumption of all refined sugars in the USA, matching a worldwide trend [3]. Current international dietary guidelines advise people to reduce their consumption of sugar, and more particularly free sugars, which comprise sugars (monosaccharides and disaccharides) added to foods or drinks by the manufacturer, cook or consumer, and sugars naturally present in honey, syrups and unsweetened fruit juices [4]. While in children a high consumption of sugar-sweetened beverages [5–7] and fruit juice [7, 8] has been linked to asthma, and particularly atopic asthma [7], the relation between total maternal consumption of free sugar during pregnancy and respiratory and atopic outcomes in the offspring has not been studied. One ecological study reported a correlation between perinatal consumption of sugar and severe childhood asthma symptoms [9], but could not specifically address maternal sugar intake in pregnancy. A recent Danish birth cohort study investigated the relation between soft drink consumption, but not total free sugar intake, during pregnancy and childhood asthma and allergic rhinitis [10].

We have investigated whether a high intake of free sugar in pregnancy is associated with adverse respiratory and atopic outcomes in the offspring in a large population-based UK birth cohort.

## Methods

### Participants

The Avon Longitudinal Study of Parents and Children (ALSPAC) is a population-based birth cohort that recruited 14 541 predominantly white pregnant women resident in Avon, UK with expected dates of delivery from April 1, 1991 to December 31, 1992. These pregnancies resulted in 13 972 singleton or twin children who were alive at 1 year of age. The cohort has been followed since birth with annual questionnaires and, since age 7 years, with objective measures in annual research clinics. The study protocol has been described previously [11, 12] and further information can be found at www.alspac.bris.ac.uk, which contains details of all the data that are available (www.bris.ac.uk/alspac/researchers/data-access/data-dictionary/). Ethics approval was obtained from the ALSPAC Ethics and Law Committee (IRB 00003312) and the Local National Health Service Research Ethics Committees.

### Exposure assessment

Data on maternal diet in pregnancy were collected by a food frequency questionnaire (FFQ) at 32 weeks gestation, covering all the main foods consumed in Britain [13]. The questionnaire asked about their current weekly frequency of consumption of 43 food groups and food items. More detailed questions were asked about daily consumption of a further eight basic foods (including sugar, coffee and tea). The FFQ was used to estimate total energy intake and daily nutrient intake, by multiplying the daily frequency of consumption of a food by the nutrient content [14] of a standard portion [15] of that food, and summing this for all the foods consumed. In this way free sugar intake was estimated. Free sugar does not include lactose when naturally present in milk and milk products or the sugars contained within the cellular structure of fruits and vegetables. Information on the child's free sugar consumption at age 3 years, as well as maternal and paternal sugar consumption at 4 years post-partum, was collected by a similar FFQ.

Information from a questionnaire at recruitment and from the obstetric records was used to classify women into four mutually exclusive categories: no evidence of glycosuria or diabetes, existing diabetes mellitus before the pregnancy, gestational diabetes and persistent glycosuria during pregnancy. For the purposes of analysis we combined the last three categories to create a binary maternal “diabetes” variable (see supplementary material for further details).

### Outcome assessment

#### Binary variables

Current doctor-diagnosed asthma was defined in children at age 7.5 years (primary outcome) if mothers responded positively to the question “Has a doctor *ever* actually *said* that your study child has asthma?” and to one or both of the questions “Has your child had any of the following in the past 12 months: wheezing with whistling; asthma?”

Current wheezing, eczema and hay fever in children at age 7.5 years were defined by a positive answer to the question: “Has your child had any of the following in the past 12 months: wheezing with whistling; eczema; hay fever?”

Atopy at age 7 years was defined as a positive reaction (maximum diameter of any detectable weal) to *Dermatophagoides* *pteronyssinus*, cat or grass (after subtracting positive saline reactions from histamine and allergen weals, and excluding children unreactive to 1% histamine).

#### Categorical variables

Children were further classified, *post hoc*, according to their asthmatic/atopic status, thus defining a four-category variable (no atopy or asthma, atopy only, nonatopic asthma and atopic asthma), and according to their number of positive reactions to cat, grass and dust mite allergens (n=0, 1 and ≥2).

Data on child's asthma status at age 7 and 14 years were used to derive asthma status phenotypes between age 7 and 14 years (none, remitting, incident and persisting) [16].

#### Continuous variables

Serum total IgE (kU·L^−1^) was measured by fluoroimmunoassay using the Pharmacia UNICAP system (Pharmacia and Upjohn Diagnostics, Uppsala, Sweden).

Lung function was measured by spirometry (Vitalograph 2120; Vitalograph, Maids Moreton, UK) at age 8.5 years after withholding short-acting bronchodilators for at least 6 h and long-acting bronchodilators and theophyllines for at least 24 h. The best of three reproducible flow–volume curves was used to measure forced expiratory volume in 1 s (FEV_1_), forced vital capacity (FVC) and maximal mid-expiratory flow (forced expiratory flow at 25–75% of FVC (FEF_25–75_)), which were further transformed to age-, height- and sex-adjusted standard deviation units [17]. The tests adhered to American Thoracic Society (ATS) criteria for standardisation and reproducibility of flow–volume measurement [18], with the exception of ATS recommendations for duration of expiration [19]; as many children did not fulfil forced expiratory time >6 s end-of-test criteria, a minimal volume change over the final 1 s was used.

### Potential confounders

We selected potential confounding factors which are known (from existing literature) to be associated with one or more of the outcomes of interest [20]. These included maternal age at delivery, sex of child, multiple pregnancy, season of birth, maternal history of atopic diseases (hay fever, asthma, eczema, allergies, or attacks of wheezing with whistling on the chest or attacks of breathlessness in the past 2 years), parity, highest educational qualification, housing tenure, financial difficulties, ethnicity, breastfeeding duration and maternal factors during pregnancy (smoking status, anxiety score (Crown–Crisp Experiential Index), paracetamol use, antibiotic use, infections (urinary infection, influenza, rubella, thrush, genital herpes, other), supplement use and total energy intake (kJ·day^−1^)). Smoking status was categorised as the maximum exposure during pregnancy (never, passive smoking only, 1–9, 10–19 and ≥20 cigarettes per day).

### Statistical analyses

We compared the distributions of child and maternal variables across maternal free sugar intake quintiles using F-statistics for differences in continuous variables and Chi-squared tests for differences in categorical variables. Logistic regression, multinomial logistic regression and linear regression were used to analyse relations between maternal free sugar intake in pregnancy and binary, categorical and continuous outcomes, respectively. After log-transforming total IgE, linear regression was used to estimate geometric mean ratios for IgE; confidence limits were calculated using Huber variances. We analysed free sugar intake in quintiles: 1) as a categorical variable using the lowest quintile as reference to allow for a nonlinear pattern of association and 2) as a continuous variable to test for linear trend (*i.e.* per quintile effect). For all regression analyses, two stages of adjustment were used. In Model 1 we adjusted for total energy intake only. In Model 2 we adjusted additionally for all potential confounders listed above.

When evidence for associations persisted, we considered other factors which can be considered either as potential confounders or potential mediators of associations between maternal free sugar intake in pregnancy and childhood outcomes, *i.e.* prematurity [21, 22], impaired fetal growth [21, 23], maternal obesity and weight gain [24–26], and offspring obesity [27, 28]. We therefore adjusted additionally for maternal pre-pregnancy body mass index (BMI) (self-reported), gestational age at delivery, birthweight, maternal weight gain during pregnancy (all abstracted from obstetric records) and child's BMI at age 7 years (based on measured height and weight at clinic) (see supplementary figure E1 showing a directed acyclic graph). In order to assess confounding arising from other dimensions of diet, we additionally adjusted separately for maternal intake of vitamin E, zinc, selenium, *n*-3 polyunsaturated fatty acids (PUFAs), *n*-6 PUFAs, and total fruits and vegetables in pregnancy [1, 29–31].

To investigate confounding by post-natal sugar intake, we adjusted additionally for child's sugar intake at age 3 years. In order to investigate potential unmeasured confounding by genetic or shared environmental or lifestyle factors, we used a parental comparison approach, whereby effect estimates for maternal sugar intake in pregnancy were compared with effect estimates for maternal and paternal sugar intake after pregnancy. If there is a causal intrauterine effect, one would expect a stronger association with maternal intake in pregnancy than with maternal post-natal intake or paternal intake (the latter two exposures cannot have a direct biological effect on offspring asthma risk) (see further details in the supplementary material) [32, 33].

As sensitivity analyses, we repeated analyses after exclusion of mothers with implausible energy intakes (<2500 or >25 000 kJ·day^−1^ [34]) and after exclusion of mothers with diabetes (whose offspring will have experienced high fetal exposure to glucose). To correct for potential loss to follow-up bias, we used inverse probability weighting and assigned to each woman a weight that was the inverse of the probability of her selection for given values of covariates (see further details in the supplementary material) [35]. All statistical analyses were carried out using Stata version 12.1 (StataCorp, College Station, TX, USA).

## Results

Of the 13 972 singleton or twin children alive at 1 year of age, information on maternal diet was available for 12 078, of whom there was information on at least one of the outcomes of interest for 8956 (supplementary figure E2). Characteristics of the 8956 mother–child pairs who were included in the analyses and those of the 3122 mother–child pairs with information on maternal diet who were excluded because of incomplete outcome data are compared in supplementary table E1. Among children with available information, 12.2% had current doctor-diagnosed asthma, 10.7% had current wheezing with whistling, 8.8% had current hay fever, 16.2% had current eczema, 21.5% had atopy and 61.8% did not have any of these five outcomes.

**FIGURE 1:**
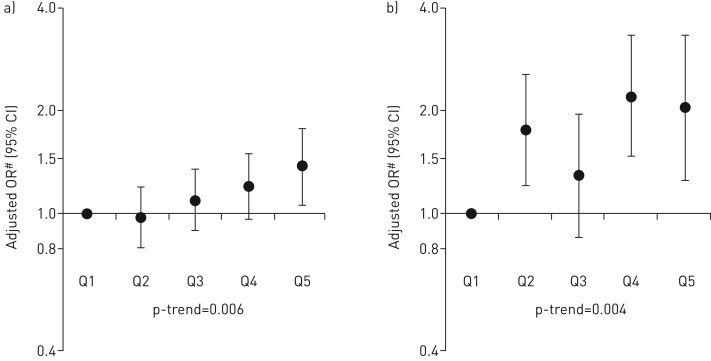
Summary of the main findings for the associations between maternal free sugar intake during pregnancy and childhood outcomes: a) atopy (n=6117) and b) atopic asthma (n=5228). Q: quintile. ^#^: controlling for energy intake, smoking, infections, supplements, antibiotics and paracetamol use during pregnancy; maternal educational level, housing tenure, financial difficulties, ethnicity, age, parity, history of atopic diseases, anxiety, sex of child, season of birth, multiple pregnancy and breastfeeding duration.

Maternal characteristics which differed across quintiles of free sugar intake during pregnancy included age, parity, pregnancy size, season of birth, breastfeeding duration, educational level, ethnicity, housing tenure, financial difficulties, anxiety level, tobacco exposure and infection during pregnancy. Women in the highest quintile of total sugar intake during pregnancy had a lower pre-pregnancy BMI, higher total energy intake and gained more weight during pregnancy than women in the lowest quintile. Their offspring were more likely to have weighed less at birth and to have had a lower BMI at age 7 years (table 1). After adjustment for potential confounders, there was weak evidence for positive associations between maternal free sugar intake in pregnancy and childhood doctor-diagnosed asthma and childhood wheeze (OR comparing highest *versus* lowest quintile 1.31, 95% CI 0.98–1.75; per quintile p*-*trend=0.09 and 1.42, 95% CI 1.05–1.92; per quintile p*-*trend=0.08, respectively), and stronger evidence for a positive association with atopy at age 7 years (OR 1.38, 95% CI 1.06–1.78; per quintile p*-*trend=0.006) (table 2). There was no association with eczema, hay fever, total IgE, FEV_1,_ FVC or FEF_25–75_ (table 2 and supplementary table E2). *Post hoc* analysis showed a positive association between maternal intake of free sugar and atopic asthma (OR 2.01, 95% CI 1.23–3.29; per quintile p*-*trend=0.004) (table 3). The main positive findings of our study are summarised in figure 1.

**TABLE 1:** Characteristics of mothers and offspring who had information on at least one of the outcomes of interest (wheeze, asthma, atopy, eczema, hay fever, total IgE and lung function) by maternal free sugar intake during pregnancy^#^

	**Free sugar intake g·day^−1^**					**p-value**
	**Quintile 1** **(1.6–34.0)**	**Quintile 2** **(34.0–46.6)**	**Quintile 3** **(46.6–60.8)**	**Quintile 4** **(60.8–82.4)**	**Quintile 5** **(82.4–345.1)**	
**Mother's age years**	29.4±4.7	29.1±4.5	29.2±4.6	28.9±4.5	27.9±4.7	<0.001
**Parity**						
0	43.2	46.1	48.6	44.9	44.0	0.001
1	35.4	37.2	35.1	36.3	35.9	
≥2	21.4	16.7	16.3	18.8	20.0	
**Sex of child**						
Male	51.2	52.3	50.5	52.3	49.5	0.39
Female	48.8	47.7	49.5	47.7	50.5	
**Multiple pregnancy**						
Singleton	97.3	96.7	98.0	98.3	97.3	0.01
Twin	2.7	3.3	2.0	1.7	2.7	
**Season of birth**						
Winter	15.0	17.6	16.6	15.7	16.0	<0.001
Spring	23.6	25.3	26.8	29.1	30.7	
Summer	33.1	29.3	31.8	28.9	27.1	
Autumn	28.3	27.8	24.9	26.4	26.2	
**Breastfeeding duration months**						
Never	21.3	18.9	17.5	22.1	27.1	<0.001
<3	34.0	30.0	30.1	29.2	34.2	
3–6	13.1	14.9	14.6	13.3	12.8	
≥6	31.5	36.3	37.4	35.4	25.9	
**Mother's educational level**						
Certificate of Secondary Education	16.7	12.9	13.4	14.5	20.4	<0.001
Vocational	8.6	9.2	8.1	8.5	10.8	
Ordinary level	36.2	35.0	32.8	35.1	38.8	
Advanced level	24.6	26.6	26.3	26.0	21.2	
Degree	14.0	16.2	19.4	15.9	8.9	
**Maternal ethnicity**						
White	97.8	97.2	98.5	98.4	98.8	0.003
Non-white	2.2	2.8	1.5	1.6	1.2	
**Housing tenure**						
Owned/mortgaged	82.6	86.5	86.5	85.1	76.8	<0.001
Council rented	9.9	7.2	6.6	8.8	15.8	
Non-council rented	7.5	6.3	7.0	6.1	7.5	
**Financial difficulties**						
No	82.6	82.8	84.3	85.3	78.8	<0.001
Yes	17.4	17.2	15.7	14.7	21.2	
**Maternal history of atopic diseases**						
No	32.2	32.0	33.7	31.4	28.9	0.05
Yes	67.8	68.0	66.3	68.6	71.1	
**Maternal anxiety score during pregnancy**						
0–9	24.0	23.4	23.1	20.8	14.3	<0.001
10–14	25.1	27.5	25.7	26.1	24.3	
15–20	24.4	25.1	27.3	27.1	25.4	
≥20	26.5	24.1	24.0	26.1	36.1	
**Maximum maternal tobacco exposure cigarettes·day^−1^**						
None	27.8	27.6	28.9	28.2	19.2	<0.001
Passive only	45.4	48.5	47.3	46.0	42.3	
1–9	7.6	7.7	8.1	8.0	8.1	
10–19	12.5	10.1	9.4	10.1	15.2	
≥20	6.7	6.2	6.2	7.7	15.2	
**Maternal paracetamol use during pregnancy**						
No	38.0	38.0	37.7	38.0	36.4	0.86
Yes	62.0	62.0	62.3	62.0	63.6	
**Maternal antibiotic use during pregnancy**						
No	83.7	85.4	84.0	83.7	82.9	0.34
Yes	16.3	14.6	16.0	16.3	17.1	
**Maternal supplement use during pregnancy**						
No	44.5	43.1	44.4	41.9	41.6	0.26
Yes	55.5	56.9	55.6	58.1	58.4	
**Maternal infections during pregnancy**						
No	57.2	55.1	55.8	53.3	49.3	<0.001
Yes	42.8	44.9	44.2	46.7	50.7	
**Total energy intake kJ·day**^−1^	5567±1376	6499±1326	7166±1360	7931±1488	9317±1994	<0.001
**Maternal pre-pregnancy BMI kg·m^−2^**						
<18.50	2.7	2.8	4.4	4.0	8.1	<0.001
18.50–24.99	70.8	74.3	77.7	76.7	77.4	
25.00–29.99	17.7	17.1	13.5	14.8	11.6	
≥30.00	8.8	5.8	4.4	4.5	2.9	
**Birthweight g**						
<2500	4.2	4.0	3.6	4.3	5.6	0.02
2500–2999	14.2	13.2	13.9	12.8	15.2	
3000–3499	34.8	34.9	35.1	36.7	35.6	
3500–3999	32.2	33.6	33.5	33.6	33.0	
≥4000	14.7	14.4	13.8	12.6	11.0	
**Gestational age weeks**	39.4±1.8	39.5±1.7	39.5±1.7	39.5±1.8	39.4±1.9	0.28
**Child's BMI at age 7** **years kg·m^−2^**						
<15.00	25.8	26.3	30.3	27.7	30.7	<0.001
15.00–17.49	51.9	52.3	51.2	54.4	52.6	
17.50–20.49	17.2	16.6	14.2	14.3	13.6	
≥20.50	5.2	4.8	4.3	3.5	3.1	
**Maternal weight gain during pregnancy**						
Quartile 1	30.3	23.7	24.5	23.5	24.5	<0.001
Quartile 2	24.3	25.0	25.1	24.7	25.0	
Quartile 3	24.2	27.0	26.3	26.4	23.4	
Quartile 4	21.3	24.3	24.1	25.4	27.1	

Data are presented as mean±sd or %, unless otherwise stated. BMI: body mass index. ^#^: n=8956.

**TABLE 2:** Associations between maternal free sugar intake during pregnancy and asthma, wheeze, eczema, hay fever and atopy in the offspring

	**Maternal free sugar intake during pregnancy**						**p*-*trend**
	**Quintile 1**	**Quintile 2**	**Quintile 3**	**Quintile 4**	**Quintile 5**	**Per quintile**	
**Asthma n=7677**							
Adjusted OR^#^ (95% CI)	1.00	1.14 (0.91–1.41)	0.97 (0.77–1.23)	1.22 (0.96–1.56)	1.37 (1.04–1.81)	1.07 (1.00–1.14)	0.04
Adjusted OR^¶^ (95% CI)	1.00	1.18 (0.94–1.47)	1.02 (0.80–1.29)	1.24 (0.97–1.58)	1.31 (0.98–1.75)	1.06 (0.99–1.13)	0.09
**Wheeze n=7762**							
Adjusted OR^#^ (95% CI)	1.00	1.21 (0.96–1.52)	1.05 (0.82–1.35)	1.19 (0.92–1.54)	1.38 (1.03–1.85)	1.06 (0.99–1.13)	0.09
Adjusted OR^¶^ (95% CI)	1.00	1.25 (0.99–1.58)	1.11 (0.86–1.42)	1.22 (0.94–1.59)	1.42 (1.05–1.92)	1.06 (0.99–1.14)	0.08
**Eczema n=7748**							
Adjusted OR^#^ (95% CI)	1.00	1.19 (0.98–1.44)	1.13 (0.93–1.38)	1.05 (0.84–1.30)	0.91 (0.70–1.17)	0.97 (0.92–1.03)	0.33
Adjusted OR^¶^ (95% CI)	1.00	1.17 (0.96–1.42)	1.15 (0.94–1.41)	1.07 (0.86–1.33)	0.97 (0.74–1.26)	0.99 (0.93–1.05)	0.70
**Hay fever n=7728**							
Adjusted OR^#^ (95% CI)	1.00	1.07 (0.82–1.38)	1.25 (0.96–1.63)	1.23 (0.93–1.63)	1.22 (0.88–1.69)	1.06 (0.98–1.14)	0.13
Adjusted OR^¶^ (95% CI)	1.00	1.03 (0.79–1.33)	1.24 (0.95–1.62)	1.21 (0.91–1.60)	1.25 (0.89–1.75)	1.07 (0.99–1.15)	0.10
**Atopy n=6117**							
Adjusted OR^#^ (95% CI)	1.00	0.99 (0.81–1.21)	1.09 (0.89–1.34)	1.19 (0.96–1.47)	1.24 (0.97–1.60)	1.06 (1.01–1.13)	0.03
Adjusted OR^¶^ (95% CI)	1.00	0.98 (0.80–1.20)	1.10 (0.89–1.35)	1.20 (0.96–1.49)	1.38 (1.06–1.78)	1.09 (1.02–1.15)	0.006

^#^: controlling for energy intake; ^¶^: controlling for energy intake, smoking, infections, supplements, antibiotics and paracetamol use during pregnancy; maternal educational level, housing tenure, financial difficulties, ethnicity, age, parity, history of atopic diseases, anxiety, sex of child, season of birth, multiple pregnancy and breastfeeding duration.

**TABLE 3:** Associations between maternal free sugar intake during pregnancy and atopy without asthma, nonatopic and atopic asthma^#^ in the offspring

	**Maternal free sugar intake during pregnancy**						**p*-*trend**
	**Quintile 1**	**Quintile 2**	**Quintile 3**	**Quintile 4**	**Quintile 5**	**Per quintile**	
**Atopy without asthma n=794**							
Adjusted OR^¶^ (95% CI)	1.00	0.78 (0.61–1.01)	1.05 (0.82–1.34)	0.96 (0.73–1.25)	1.09 (0.80–1.50)	1.04 (0.97–1.12)	0.30
Adjusted OR^+^ (95% CI)	1.00	0.77 (0.60–0.99)	1.05 (0.82–1.34)	0.95 (0.72–1.25)	1.17 (0.85–1.62)	1.05 (0.98–1.13)	0.19
**Nonatopic asthma n=301**							
Adjusted OR^¶^ (95% CI)	1.00	0.82 (0.57–1.16)	0.67 (0.46–0.99)	0.77 (0.52–1.16)	0.83 (0.52–1.34)	0.95 (0.85–1.06)	0.34
Adjusted OR^+^ (95% CI)	1.00	0.87 (0.61–1.25)	0.73 (0.49–1.08)	0.78 (0.52–1.18)	0.71 (0.43–1.15)	0.92 (0.82–1.03)	0.14
**Atopic asthma n=337**							
Adjusted OR^¶^ (95% CI)	1.00	1.66 (1.14–2.41)	1.17 (0.78–1.77)	2.09 (1.39–3.14)	1.79 (1.11–2.90)	1.14 (1.03–1.27)	0.01
Adjusted OR^+^ (95% CI)	1.00	1.75 (1.20–2.56)	1.27 (0.84–1.93)	2.18 (1.45–3.30)	2.01 (1.23–3.29)	1.17 (1.05–1.30)	0.004

^#^: no atopy or asthma was considered as baseline category (n=3796); ^¶^: controlling for energy intake; ^+^: controlling for energy intake, smoking, infections, supplements, antibiotics and paracetamol use during pregnancy; maternal educational level, housing tenure, financial difficulties, ethnicity, age, parity, history of atopic diseases, anxiety, sex of child, season of birth, multiple pregnancy and breastfeeding duration.

### Further investigation of potential confounding/mediation of main findings and sensitivity analyses

Additional separate adjustment for maternal pre-pregnancy BMI, gestational age at delivery, birthweight, maternal weight gain during pregnancy and child's BMI at age 7 years did not substantially alter the main findings and therefore no further formal mediation analysis was conducted (supplementary table E3). Additional separate adjustment for maternal intake of vitamin E, zinc, selenium, n-3 PUFAs, n-6 PUFAs, and total fruits and vegetables in pregnancy did not substantially alter the main findings (data not shown), nor did additional separate adjustment for child's free sugar intake at age 3 years (supplementary table E3). The latter exposure was not associated with any outcome (data not shown).

In subsets of the cohort with complete data for paternal (respectively, maternal) free sugar intake after pregnancy, no association was found between paternal (respectively, maternal) free sugar intake after pregnancy and childhood atopy or atopic asthma. The significant associations of maternal free sugar intake during pregnancy with childhood atopy and atopic asthma remained, unattenuated, on mutual adjustment for paternal (respectively, maternal) post-natal exposure (table 4 and supplementary table E4, respectively).

**TABLE 4:** Comparison of associations of childhood atopy and atopic asthma with maternal free sugar intake during pregnancy *versus* paternal intake after pregnancy

	**Free sugar intake**						**p*-*trend**
	**Quintile 1**	**Quintile 2**	**Quintile 3**	**Quintile 4**	**Quintile 5**	**Per quintile**	
**Atopy n=3063**							
Maternal free sugar intake during pregnancy							
Adjusted OR^#^ (95% CI)	1.00	1.04 (0.78–1.38)	1.16 (0.87–1.55)	1.33 (0.98–1.82)	1.64 (1.14–2.37)	1.13 (1.04–1.23)	0.004
Adjusted OR^¶^ (95% CI)	1.00	1.02 (0.77–1.36)	1.15 (0.86–1.53)	1.31 (0.96–1.79)	1.61 (1.11–2.33)	1.13 (1.04–1.22)	0.005
Paternal free sugar intake after pregnancy							
Adjusted OR^#^ (95% CI)	1.00	1.12 (0.84–1.50)	1.28 (0.95–1.73)	1.36 (0.99–1.86)	1.16 (0.81–1.68)	1.05 (0.97–1.14)	0.22
Adjusted OR^¶^ (95% CI)	1.00	1.10 (0.82–1.47)	1.25 (0.93–1.69)	1.30 (0.95–1.78)	1.10 (0.76–1.59)	1.04 (0.96–1.13)	0.34
**Atopic asthma n=2830**							
Maternal free sugar intake during pregnancy							
Adjusted OR^#^ (95% CI)	1.00	2.11 (1.26–3.52)	1.18 (0.66–2.11)	2.82 (1.60–4.96)	2.01 (1.01–4.00)	1.17 (1.02–1.36)	0.03
Adjusted OR^¶^ (95% CI)	1.00	2.13 (1.27–3.57)	1.17 (0.65–2.11)	2.80 (1.58–4.94)	1.96 (0.98–3.93)	1.17 (1.01–1.35)	0.04
Paternal free sugar intake after pregnancy							
Adjusted OR^#^ (95% CI)	1.00	0.99 (0.60–1.62)	1.26 (0.75–2.11)	1.47 (0.87–2.48)	0.92 (0.48–1.77)	1.04 (0.90–1.21)	0.55
Adjusted OR^¶^ (95% CI)	1.00	0.96 (0.59–1.59)	1.23 (0.73–2.06)	1.39 (0.82–2.35)	0.87 (0.45–1.69)	1.03 (0.89–1.19)	0.72

^#^: controlling only for previously mentioned potential confounders; ^¶^: mutually adjusting for maternal free sugar intake during pregnancy and paternal free sugar intake after pregnancy, in addition to previously mentioned potential confounders

When we analysed the association between maternal free sugar intake and the number of positive reactions to cat, grass and dust mite allergens, we observed a stronger association for children with two or more positive reactions (table 5). We studied associations between maternal free sugar intake in pregnancy and childhood asthma status phenotypes, and did not observe any association (supplementary table E5).

**TABLE 5:** Associations between maternal free sugar intake in pregnancy and number of positive skin prick tests (SPTs)^#^

**Free sugar**	**1 positive SPT**^¶^		**≥2 positive SPTs**^+^	
	**Adjusted OR^§^** **(95% CI)**	**p-trend**	**Adjusted OR^§^** **(95% CI)**	**p-trend**
**Quintile 1**	1.00		1.00	
**Quintile 2**	0.87 (0.68–1.12)		1.17 (0.87–1.57)	
**Quintile 3**	1.07 (0.83–1.37)		1.14 (0.84–1.56)	
**Quintile 4**	1.09 (0.83–1.43)		1.37 (0.99–1.90)	
**Quintile 5**	1.19 (0.86–1.63)		1.73 (1.18–2.52)	
**Per quintile**	1.06 (0.98–1.14)	0.12	1.13 (1.03–1.23)	0.006

^#^: no positive SPT was considered as baseline category (n=4797); ^¶^: n=783; ^+^: n=531; ^§^: controlling for energy intake, smoking, infections, supplements, antibiotics and paracetamol use during pregnancy; maternal educational level, housing tenure, financial difficulties, ethnicity, age, parity, history of atopic diseases, anxiety, sex of child, season of birth, multiple pregnancy and breastfeeding duration.

The exclusion of 17 women with implausible energy intake estimates did not alter the main results nor did exclusion of mothers with diabetes. Maternal “diabetes” was not associated with any respiratory or atopic outcome, but was associated with higher birthweight. The inverse probability weighting analysis also produced similar results (data not shown).

## Discussion

In this population-based birth cohort study, we found that a higher maternal intake of free sugar during pregnancy was associated with an increased risk of atopy and atopic asthma in the offspring, independently of sugar intake in early childhood.

To the best of our knowledge, these are novel findings. While a previous ecological study reported a positive correlation between perinatal consumption of sugar and severe childhood asthma symptoms [9], the limitations of ecological studies for inferring causality are well known (not least because of the high likelihood of confounding) [36]. Furthermore, that study was unable to specifically investigate the potential role of maternal intake of sugar during pregnancy nor the specific role of free sugar. Our longitudinal findings linking maternal free sugar intake in pregnancy to childhood atopy and atopic asthma extend the ecological results and allow stronger causal inference. Interestingly, the findings for atopy became stronger when we examined the association with multiple sensitisation. Although previous cross-sectional studies have reported a positive association between childhood consumption of sugar-containing drinks, including fruit juice, and asthma [5–8], intake of free sugar in early childhood in our study was not associated with any respiratory or atopic outcome.

### Mechanisms

We speculate that high maternal fructose consumption may underlie the positive associations between maternal intake of free sugar and childhood atopy and atopic asthma. Fructose, which is a major component of added sugars, and is present naturally in fruit juice and in sweetened drinks as added sucrose (ratio of fructose/glucose 50/50%) or isolated fructose, has been mooted as driving previous cross-sectional findings linking sugar-containing beverage consumption to asthma in children [7, 8]. Fructose consumption, in the form of high fructose corn syrup (ratio of fructose/glucose 60/40%), increased from near 0% to near 30% of per capita consumption of refined sugars in the USA between 1970 and 2000, whereas the consumption of sucrose and glucose declined or remained constant [3].

A prospective randomised controlled trial in adults showed that dietary sugar, and especially fructose, increased levels of C-reactive protein [37]. Fructose also causes generation of uric acid [38], and experimental evidence in mice suggests that uric acid may be an essential initiator and amplifier of T-helper cell type 2 (Th2) immunity and allergic inflammation, through activation of inflammatory dendritic cells [39]. Alternatively, fructose might influence atopic immune responses by conditioning the gut microbiome [40, 41]. The potential of maternal diet in pregnancy to influence inception of offspring allergic airways disease through this mechanism was recently confirmed in a mouse model [42]. We therefore propose that one explanation for our main findings is that high fetal exposure to fructose may cause persistence of Th2 immune responses post-natally and allergic inflammation in the developing lung.

In contrast to a previous study which reported a link between gestational diabetes and risk of atopic eczema and atopy in early childhood [43], we found no association between maternal diabetes during pregnancy and any outcome in the offspring, although, as expected [44], maternal diabetes was associated with higher birthweight. The lack of a relation with maternal diabetes would suggest that higher fetal exposure to glucose is unlikely to explain our main findings. While high fructose consumption has been proposed as a risk factor for obesity [45], we found no evidence to suggest that the associations between maternal free sugar intake and atopy and atopic asthma in the offspring were mediated by maternal BMI, gestational weight gain or child's BMI, nor by prematurity or low birthweight, assuming key assumptions necessary for mediation analyses were met [46, 47].

### Strengths and limitations

Strengths of the ALSPAC birth cohort include its size and population-based prospective design, rich information on numerous potential lifestyle and dietary confounders (including information on childhood free sugar intake and parental sugar intake outside of pregnancy), and detailed phenotypic outcome measurements.

Although the FFQ that we used had not been formally calibrated against other instruments such as diet diaries, it was based on the one used by Yarnell *et al.* [48], which has been validated against weighed dietary records and modified in the light of a more recent weighed dietary survey [13]. The FFQ lacked quantitative information on soft drink consumption and this will have led to underestimation of maternal free sugar intake during pregnancy. However, as misclassification of maternal free sugar intake in pregnancy is likely to have been random with respect to childhood outcomes, the strength of associations may have been underestimated. We were unable to assess associations with maternal sugar intake in early pregnancy; however, intakes in early and late pregnancy are likely to be highly correlated. We were unable to assess whether associations between maternal intake of free sugar in pregnancy and childhood atopy and atopic asthma persist beyond the age of 7 years, as no data on atopy (only data on asthma status) have been collected in ALSPAC children after the age of 7 years.

We think that confounding of the main findings by lifestyle or other aspects of maternal diet in pregnancy is unlikely, as we controlled for numerous potential confounders in the analyses, including nutrients and foods that have been previously linked to childhood asthma and atopy. Importantly, the main findings were not confounded by the offspring's free sugar intake in early childhood. While the possibility of residual confounding cannot be ruled out, the null findings for maternal and paternal free sugar intakes after pregnancy make confounding by unmeasured familial behaviours linked to sugar intake and asthma risk a less likely explanation.

As with any longitudinal study, we cannot rule out the possibility that exclusion of mother–child pairs without complete information might have biased our findings. However, it could be argued that, for our results to be totally spurious in those included in our analysis (and for the associations to be truly null in the population as a whole), associations in the excluded mother–child pairs would have to be in the opposite direction and much stronger, compared with the positive associations we reported in the included mother–child pairs, which seems extremely unlikely. Furthermore, loss to follow-up bias has been shown to only slightly modify associations in longitudinal studies, including in ALSPAC [49], and the results of our inverse probability weighting analysis [35] confirmed that loss to follow-up is unlikely to have biased our results. In view of the multiple analyses carried out and the *post hoc* nature of the findings for atopic asthma, we cannot exclude the possibility that the main findings occurred by chance; hence, they should be interpreted with caution. Given the *a priori* nature of the hypothesis being tested, and the fact that some outcomes of interest are highly correlated, it did not seem appropriate to correct for multiple testing. However, we plan to re-examine this hypothesis in another birth cohort to see if we can replicate the main findings.

### Conclusions and public health implications

We conclude that a higher maternal intake of free sugar during pregnancy may increase the risk of atopy and atopic asthma in the offspring. If these findings are replicated we would design an appropriate intervention study in pregnancy to establish or refute causality. Given the very high levels of sugar consumption currently in the West, where childhood allergy and asthma are so prevalent, confirmation of a causal link would raise exciting prospects for the primary prevention of these disorders.

## Supplementary material

10.1183/13993003.00073-2017.Supp1 **Please note:** supplementary material is not edited by the Editorial Office, and is uploaded as it has been supplied by the author.
Supplementary material ERJ-00073-2017_Supplement

## Disclosures

10.1183/13993003.00073-2017.Supp2 A. Bédard ERJ-00073-2017_Bedard
A.J. Henderson ERJ-00073-2017_Henderson
