# Supplementary material for: Maternal intake of sugar during pregnancy and childhood respiratory and atopic outcomes
Source: Eur Respir J. 2017 Jul 6;50(1):1700073. doi: 10.1183/13993003.00073-2017 (PMC5540678; doi:10.1183/13993003.00073-2017)
Supplement: Supplementary file 1 [file ERJ-00073-2017_Supplement.pdf]

Online data supplement

Maternal intake of sugar during pregnancy and childhood respiratory and atopic outcomes

Annabelle Bédard[1], PhD, Kate Northstone[2,3], PhD, A John Henderson[3*], MD, Seif O Shaheen[1*], PhD

[1]Centre for Primary Care and Public Health, Barts and The London School of Medicine and Dentistry, London, Queen Mary University of London, UK

[2]NIHR CLAHRC West, Bristol, UK

[3] School of Social and Community Medicine, University of Bristol, UK

[*] Joint senior authors

Supplementary methods

Definition of existing diabetes, gestational diabetes and glycosuria

At recruitment, women were asked by questionnaire about existing diabetes and any previous history of gestational diabetes. Information on glycosuria (recorded as none, trace, +, ++, +++ or more) was abstracted from the records of each antenatal clinic visit made by the woman (median number, 14 per woman). The practice in the UK at the time was for all women to be offered urine tests for glycosuria at each antenatal clinic visit. Universal screening of women with a random or fasting blood glucose level or with an oral glucose tolerance test was not undertaken, and diagnostic tests for gestational diabetes will only have been undertaken in women with established risk factors (family history, previous history of gestational diabetes or macrosomic birth, South Asian ethnicity) or persistent glycosuria. Glycosuria was defined as a record of at least ++ (equal to 13.9 mmol/l or 250 mg/100 ml according to the manufacturer (Bayer)) on at least two occasions at any time during the pregnancy (1). Women were classified into one of four mutually exclusive categories: no evidence of glycosuria or diabetes; existing diabetes before the pregnancy; gestational diabetes (i.e. a diagnosis in the medical records of gestational diabetes in any woman with no history of existing diabetes); and persistent glycosuria (i.e. ++ glycosuria on two occasions in women with no evidence of existing or gestational diabetes).

Parental comparison approach

Proof of concept has been illustrated in ALSPAC with maternal smoking in pregnancy which is strongly associated with lower offspring birth weight, whereas paternal smoking is only weakly associated (and not associated at all after mutual adjustment). In contrast, paternal and maternal smoking in pregnancy are similarly associated with offspring BMI, even after mutual adjustment, suggesting that these associations are non-causal and generated by confounding (2). We have also

used this approach to investigate the likely causal role of prenatal paracetamol exposure in the development of asthma in ALSPAC(3).

Inverse probability weighting

Inverse probability weighting has been proposed as a way to correct for selection bias (4). By assigning to each subject a weight that is the inverse of the probability of his/her selection based on a given set of covariates and exposure, inverse probability weighting creates a pseudo-population in which effect measures are not affected by selection bias (provided that the outcome in the uncensored subjects truly represents the outcome in the censored subjects for the same values of covariates and exposure). We used this approach by estimating for each woman, the probability of her selection for given values of covariates (ie. the characteristics for which differences between excluded and included women were found to be statistically significant, including the exposure – see online Table E1) and assigning her a weight that is the inverse of that probability.

References

E1. A. Lawlor D, Fraser A, Lindsay RS, Ness A, Dabelea D, Catalano P, Davey Smith G, Sattar N, Nelson SM. Association of existing diabetes, gestational diabetes and glycosuria in pregnancy with macrosomia and offspring body mass index, waist and fat mass in later childhood: findings from a prospective pregnancy cohort. *Diabetologia* 2010;53:89–97.

E2. Smith GD. Assessing intrauterine influences on offspring health outcomes: Can epidemiological studies yield robust findings? *Basic Clin Pharmacol Toxicol* 2008;102:245–256.

E3. Shaheen SO, Newson RB, Smith GD, Henderson AJ. Prenatal paracetamol exposure and asthma: Further evidence against confounding. *Int J Epidemiol* 2010;39:790–794.

E4. Hernán MA, Hernandez-Diaz S, Robins JM. A structural approach to selection bias. *Epidemiology* 2004;15:615–625.

Figure E1. Directed acyclic graph showing potential confounders and mediators of the associations between maternal intake of free sugar in pregnancy and offspring respiratory and atopic outcomes

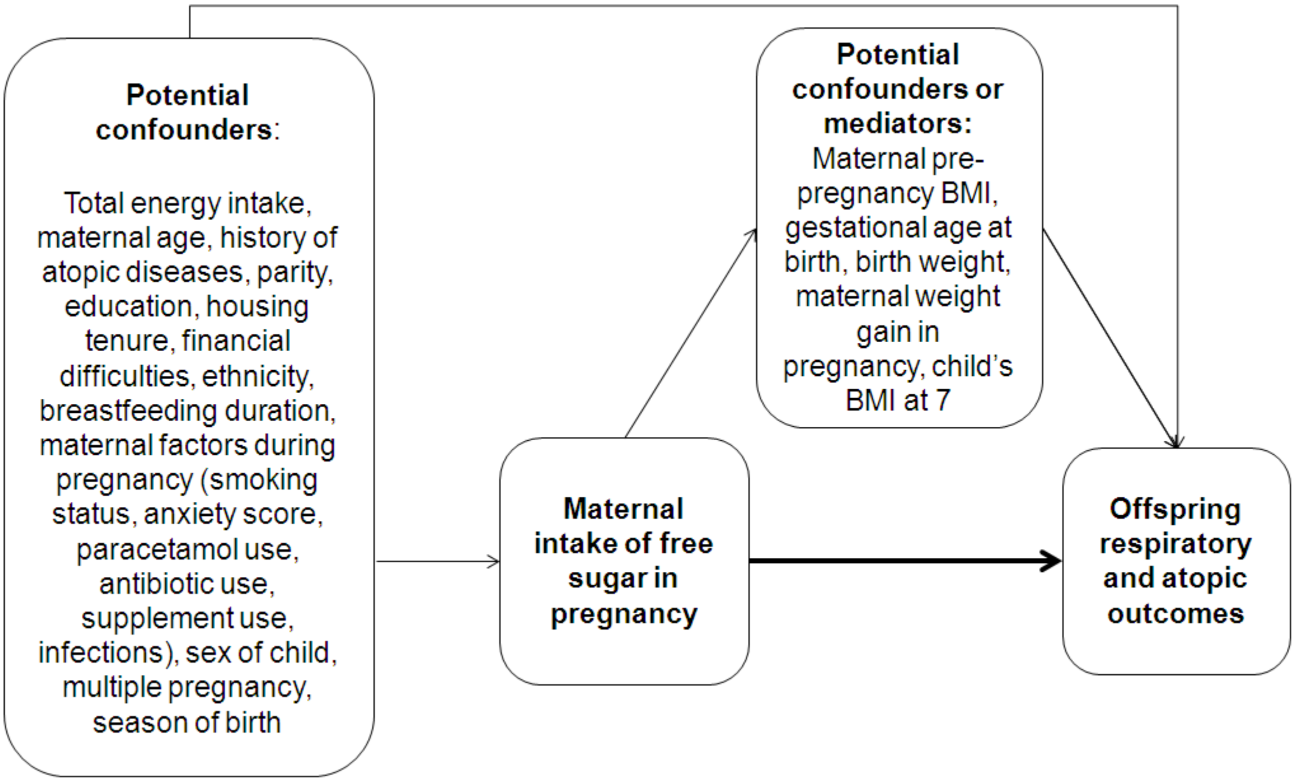

Figure E2. Participant flow

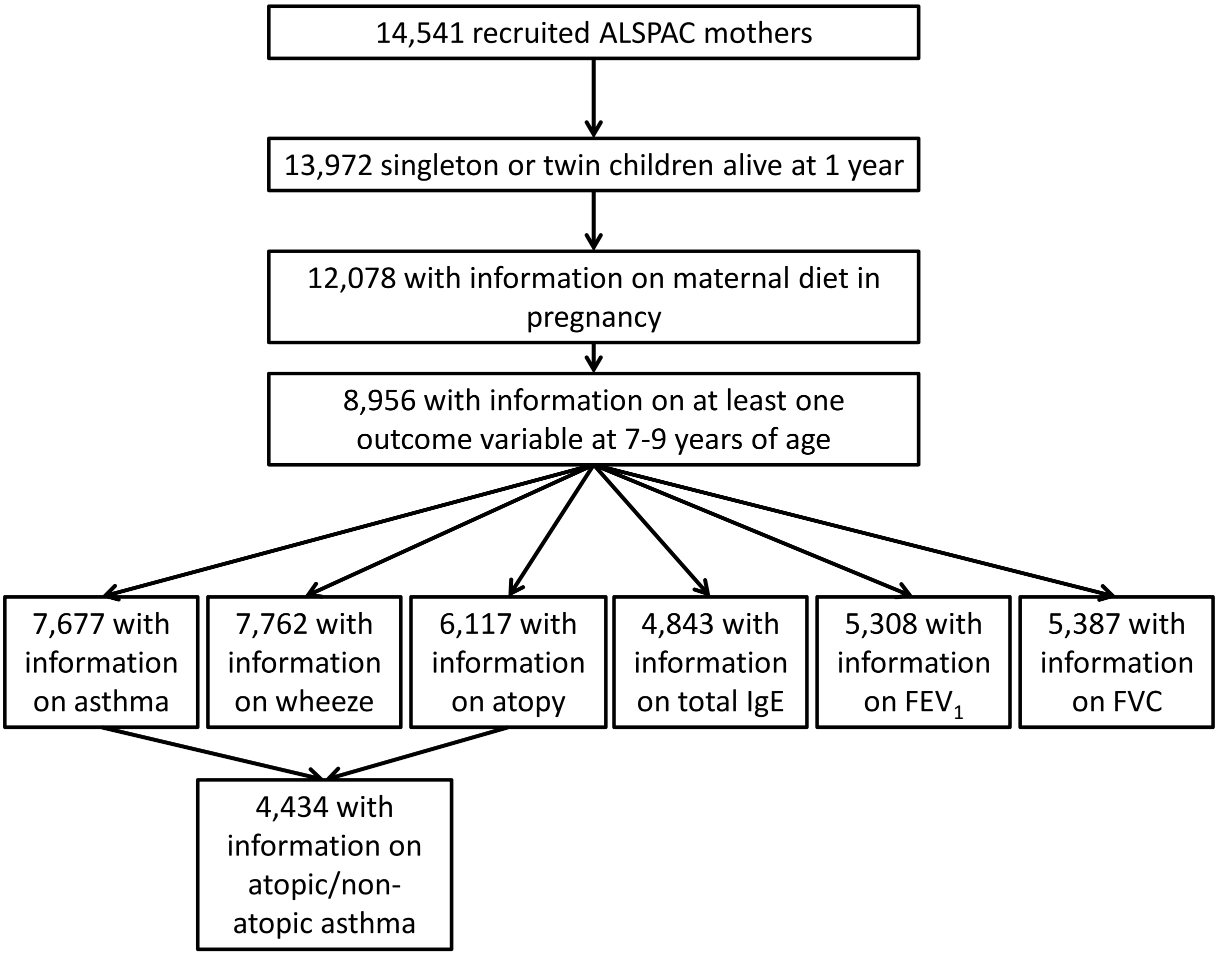

Table E1. Characteristics of mothers and offspring who were included in analyses and those who were excluded (n=12,078)

	**Included (n=8,956)**	**Excluded (n=3,122)**	***P***
**Maternal free sugar intake in pregnancy** (g/day)	59.1 (32.5)	64.5 (39.5)	< .001
**Mother's age** (years), m (sd)	28.9 (4.6)	26.5 (5.1)	< .001
**Parity,** %			
0	45.4	43.0	
1	36.0	34.3	< .001
≥2	18.6	22.8	
**Sex of child,** %			
Male	51.2	52.1	0.38
Female	48.8	47.9	
**Multiple pregnancy,** %			
Singleton	97.5	97.2	0.27
Twin	2.5	2.8	
**Season of birth**, %			
Winter	16.2	16.0	
Spring	27.0	26.5	0.60
Summer	30.1	31.4	
Autumn	26.7	26.2	
**Breastfeeding duration**, %			
Never	21.2	35.6	
<3 months	31.5	32.9	< .001
3-6 months	13.8	10.4	
≥6 months	33.5	21.0	
**Mother's educational level,** %			
Certificate of Secondary Education	15.5	32.8	
Vocational	9.0	12.3	< .001
Ordinary level	35.5	32.5	
Advanced level	25.0	15.6	
Degree	15.0	6.9	
**Maternal ethnicity**, %			
White	98.1	95.5	< .001
Non-white	1.9	4.5	
**Housing tenure,** %			
Owned/mortgaged	83.7	62.3	
Council rented	9.5	24.0	< .001
Non-council rented	6.9	13.7	
**Financial difficulties,** %			
No	82.9	77.1	< .001
Yes	17.1	22.9	
**Maternal history of atopic diseases,** %			
No	31.7	31.1	0.54
Yes	68.3	68.9	
**Maternal anxiety score in pregnancy,** %			

0-9	21.3	16.9	
10-14	25.8	21.4	< .001
15-20	25.8	24.7	
≥20	27.2	37.1	
**Maximum maternal tobacco exposure,** %			
None	26.5	17.5	
Passive only	46.0	35.8	< .001
1-9 cig/day	7.9	9.6	
10-19 cig/day	11.4	20.0	
20+ cig/day	8.3	17.1	
**Maternal paracetamol use during pregnancy,** %	37.7	35.2	0.02
No	62.4	64.8	
Yes			
**Maternal antibiotic use during pregnancy,** %			
No	84.0	85.4	0.06
Yes	16.0	14.6	
**Maternal supplement use during pregnancy,** %			
No	43.1	41.1	0.05
Yes	56.9	58.9	
**Maternal infections in pregnancy,** %			
No	54.2	53.2	0.33
Yes	45.8	46.8	
**Total energy intake** (kJ/day), m (sd)	7259 (1967)	7163 (2152)	0.02
**Maternal pre-pregnancy BMI,** %			
<18.50 kg/m²	4.3	6.4	
18.50-24.99 kg/m²	75.4	72.8	< .001
25.00-29.99 kg/m²	15.0	15.1	
≥30.00 kg/m²	5.3	5.8	
**Birth weight,** %			
<2500 g	4.3	5.7	
2500-2999 g	13.8	15.1	< .001
3000-3499 g	35.4	36.6	
3500-3999 g	33.2	30.8	
≥4000 g	13.3	11.8	
**Gestational age** (weeks), m (sd)	39.5 (1.8)	39.4 (1.9)	0.04
**Child's BMI at 7,** %			
<15.00 kg/m²	28.1	31.6	
15.00-17.49 kg/m²	52.5	43.9	0.52
17.50-20.49 kg/m²	15.2	17.5	
≥20.50 kg/m²	4.2	7.0	
**Maternal weight gain during pregnancy**, %			
Quartile 1	25.3	28.4	
Quartile 2	24.8	24.4	< .001
Quartile 3	25.5	22.1	
Quartile 4	24.4	25.2	

Table E2. Associations between maternal free sugar intake during pregnancy and total IgE, FEV_1, FVC and FEF_{25-75} in the offspring

	**Maternal free sugar intake during pregnancy**						
	1st quintile	2nd quintile	3rd quintile	4th quintile	5th quintile	Per quintile	*P*-trend
**IgE** (n=4,843)							
GMR[a] (95% CI)	1.00	1.12 (0.97-1.30)	0.95 (0.82-1.11)	1.04 (0.88-1.22)	1.07 (0.88-1.30)	1.00 (0.96-1.05)	0.95
GMR[b] (95% CI)	1.00	1.15 (0.99-1.33)	0.99 (0.85-1.15)	1.10 (0.93-1.30)	1.18 (0.97-1.43)	1.02 (0.98-1.07)	0.30
**FEV_1** (n=5,308)							
β[a] (95% CI)	0.00	-0.04 (-0.11, 0.03)	0.02 (-0.05, 0.09)	0.00 (-0.07, 0.08)	-0.03 (-0.12, 0.06)	0.00 (-0.02, 0.02)	1.00
β[b] (95% CI)	0.00	-0.04 (-0.11, 0.03)	0.01 (-0.06, 0.09)	-0.00 (-0.08, 0.08)	-0.02 (-0.11, 0.07)	0.00 (-0.02, 0.02)	0.92
**FVC** (n=5,387)							
β[a] (95% CI)	0.00	-0.03 (-0.10, 0.03)	0.02 (-0.05, 0.09)	0.01 (-0.07, 0.09)	-0.07 (-0.16, 0.02)	-0.01 (-0.03, 0.01)	0.54
β[b] (95% CI)	0.00	-0.03 (-0.10, 0.03)	0.02 (-0.06, 0.09)	0.00 (-0.07, 0.08)	-0.07 (-0.16, 0.02)	-0.01 (-0.03, 0.01)	0.50
**FEF_{25-75}** (n=5,321)							
β[a] (95% CI)	0.00	-0.02 (-0.09, 0.05)	-0.03 (-0.10, 0.04)	-0.01 (-0.08, 0.07)	-0.04 (-0.13, 0.05)	-0.01 (-0.03, 0.01)	0.56
β[b] (95% CI)	0.00	-0.02 (-0.09, 0.05)	-0.03 (-0.10, 0.05)	-0.00 (-0.08, 0.08)	-0.01 (-0.10, 0.09)	0.00 (-0.02, 0.02)	0.99

GMR: geometric mean ratio

[a] Controlling for energy intake

[b] Controlling for energy intake, smoking, infections, supplements, antibiotics and paracetamol use during pregnancy; maternal educational level, housing tenure, financial difficulties, ethnicity, age, parity, history of atopic diseases, anxiety; sex of child, season of birth, multiple pregnancy, breastfeeding duration

Table E3. Associations between maternal free sugar intake during pregnancy and childhood atopy and atopic asthma further adjusted for potential mediators and child's free sugar intake at age 3 years

	Free sugar intake						
	1st quintile	2nd quintile	3rd quintile	4th quintile	5th quintile	Per quintile	*P*-trend
**Atopy** (n=6,117)							
OR[a] (95% CI)	1.00	0.97 (0.79-1.19)	1.08 (0.88-1.33)	1.18 (0.95-1.47)	1.34 (1.03-1.73)	1.08 (1.02-1.15)	0.01
OR[b] (95% CI)	1.00	0.95 (0.76-1.17)	1.04 (0.84-1.30)	1.16 (0.91-1.46)	1.39 (1.06-1.84)	1.09 (1.02-1.16)	0.01
**Atopic asthma** (n=5,228)							
OR[a] (95% CI)	1.00	1.75 (1.20, 2.57)	1.28 (0.84, 1.94)	2.17 (1.43, 3.28)	1.97 (1.20, 3.25)	1.17 (1.05, 1.30)	0.005
OR[b] (95% CI)	1.00	1.79 (1.20, 2.65)	1.33 (0.86, 2.04)	2.07 (1.34, 3.21)	2.07 (1.23, 3.48)	1.16 (1.04, 1.30)	0.01

[a] Controlling for maternal pre-pregnancy BMI, gestational age at delivery, birth weight, maternal weight gain during pregnancy, and child's BMI at 7, in addition to all the potential confounders previously mentioned
[b] Controlling for child's free sugar intake at age 3 years, in addition to all the potential confounders previously mentioned

Table E4. Comparison of associations of childhood atopy and atopic asthma with maternal free sugar intake during pregnancy vs maternal intake after pregnancy

	Free sugar intake						
	1st quintile	2nd quintile	3rd quintile	4th quintile	5th quintile	Per quintile	*P*-trend
**Atopy** (n=5,406)							
Maternal free sugar intake in pregnancy							
OR[a] (95% CI)	1.00	0.97 (0.78-1.20)	1.07 (0.86-1.33)	1.16 (0.92-1.46)	1.37 (1.04-1.80)	1.08 (1.02-1.15)	0.01
OR[b] (95% CI)	1.00	0.94 (0.75-1.16)	1.02 (0.82-1.28)	1.10 (0.86-1.41)	1.34 (0.99-1.79)	1.07 (1.00-1.14)	0.06
Maternal free sugar intake after pregnancy							
OR[a] (95% CI)	1.00	1.08 (0.87-1.35)	1.23 (0.99-1.54)	1.32 (1.04-1.67)	1.19 (0.89-1.58)	1.06 (1.00-1.13)	0.07
OR[b] (95% CI)	1.00	1.07 (0.86-1.34)	1.20 (0.95-1.51)	1.24 (0.97-1.60)	1.04 (0.77-1.42)	1.04 (0.97-1.11)	0.33
**Atopic asthma** (n=4,840)							
Maternal free sugar intake in pregnancy							
OR[a] (95% CI)	1.00	1.75 (1.18-2.59)	1.25 (0.82-1.93)	2.14 (1.40-3.28)	1.84 (1.10-3.09)	1.15 (1.03-1.28)	0.01
OR[b] (95% CI)	1.00	1.73 (1.17-2.58)	1.24 (0.80-1.93)	2.11 (1.35-3.32)	1.86 (1.06-3.24)	1.14 (1.01-1.29)	0.03
Maternal free sugar intake after pregnancy							
OR[a] (95% CI)	1.00	0.97 (0.66-1.44)	1.25 (0.85-1.86)	1.29 (0.85-1.97	1.18 (0.71-1.96)	1.07 (0.96-1.20)	0.23
OR[b] (95% CI)	1.00	0.91 (0.61-1.35)	1.11 (0.74-1.67)	1.07 (0.69-1.67)	0.94 (0.54-1.63)	1.02 (0.90-1.15)	0.80

OR: Odds ratio

[a] Controlling only for previously mentioned potential confounders

[b] Mutually adjusting for maternal free sugar intake in pregnancy and maternal free sugar intake after pregnancy, in addition to previously mentioned potential confounders

Table E5. Associations between maternal free sugar intake in pregnancy and childhood asthma status between 7 and 14 years*

	**Remittent asthma (n=675)**		**Incident asthma (n=349)**		**Persistent asthma (n=428)**	
	OR[a] (95% CI)	*P* trend	OR[a] (95% CI)	*P* trend	OR[a] (95% CI)	*P*trend
**Free sugar**						
1st quintile	1.00		1.00		1.00	
2nd quintile	1.07 (0.83, 1.39)		1.09 (0.77, 1.54)		1.38 (1.00, 1.91)	
3rd quintile	0.87 (0.66, 1.15)		1.05 (0.73, 1.51)		1.20 (0.85, 1.69)	
4th quintile	1.07 (0.80, 1.43)		0.89 (0.59, 1.33)		1.49 (1.04, 2.13)	
5th quintile	1.05 (0.75, 1.48)		1.40 (0.89, 2.19)		1.25 (0.81, 1.93)	
Per quintile	1.01 (0.93, 1.09)	0.86	1.04 (0.93, 1.15)	0.49	1.06 (0.96, 1.16)	0.24

OR: Odds ratio

* No asthma at 7 or 14 years was considered as baseline category (n=4,250)

[a] Controlling for energy intake, smoking, infections, supplements, antibiotics and paracetamol use during pregnancy; maternal educational level, housing tenure, financial difficulties, ethnicity, age, parity, history of atopic diseases, anxiety; sex of child, season of birth, multiple pregnancy, breastfeeding duration
